# Phylodynamic analysis of avian infectious bronchitis virus in South America

**DOI:** 10.1099/vir.0.000077

**Published:** 2015-06

**Authors:** Ana Marandino, Ariel Pereda, Gonzalo Tomás, Martín Hernández, Gregorio Iraola, María Isabel Craig, Diego Hernández, Alejandro Banda, Pedro Villegas, Yanina Panzera, Ruben Pérez

**Affiliations:** 1 Sección Genética Evolutiva, Instituto de Biología, Facultad de Ciencias, Universidad de la República, Iguá 4225, 11400 Montevideo, Uruguay; 2 Instituto de Virología, CICVyA, INTA-Castelar, CC 25 (1712) Castelar, Buenos Aires, Argentina; 3 Unidad de Bioinformática, Instituto Pasteur de Montevideo, 11400 Montevideo, Uruguay; 4 Poultry Research and Diagnostic Laboratory, College of Veterinary Medicine, Mississippi State University, PO Box 97813, Pearl, MS 39288, USA; 5 College of Veterinary Medicine, Poultry Diagnostic and Research Center, University of Georgia, 953 College Station Road, Athens, GA 30602-4875, USA

## Abstract

Infectious bronchitis virus (IBV) is a coronavirus of chickens that causes great economic losses to the global poultry industry. The present study focuses on South American IBVs and their genetic relationships with global strains. We obtained full-length sequences of the S1 coding region and N gene of IBV field isolates from Uruguay and Argentina, and performed Phylodynamic analysis to characterize the strains and estimate the time of the most recent common ancestor. We identified two major South American genotypes, which were here denoted South America I (SAI) and Asia/South America II (A/SAII). The SAI genotype is an exclusive South American lineage that emerged in the 1960s. The A/SAII genotype may have emerged in Asia in approximately 1995 before being introduced into South America. Both SAI and A/SAII genotype strains clearly differ from the Massachusetts strains that are included in the vaccine formulations being used in most South American countries.

Infectious bronchitis (IB) is a globally distributed avian disease that represents one of the most persistent sanitary problems to the commercial poultry industry. The intensive production of high-density bird populations promotes IB transmission and, in spite of intensive control programmes, outbreaks are extremely frequent in commercial flocks (USDA, 2014).

The aetiological agent of IB is the infectious bronchitis virus (IBV), belonging to the genus *Gammacoronavirus* within the *Coronaviridae* family (de Groot, 2012). The IBV positive-sense ssRNA genome (27.6 kb) encodes four structural proteins: the spike (S) glycoprotein, the membrane glycoprotein, the envelope protein and the phosphorylated nucleocapsid (N) protein (Stern & Sefton, 1982). The S and N proteins are the major inducers of immune response. The S glycoprotein is post-translationally cleaved, at a cleavage recognition site sequence in the amino-terminal S1 and carboxy-terminal S2 subunits by a cellular protease during viral maturation (Lai & Cavanagh, 1997). The S1 subunit contains epitopes and determinants for virus-neutralizing antibodies, cell attachment and serotype specificity (Ignjatovic & Galli, 1994). The S2 subunit anchors the S1 protein to the membrane and is involved in membrane fusion. The N protein plays a role in regulation of IBV replication (Fan *et al.*, 2005), grouping virus particles, and inducing T-cell-mediated immune responses (Collisson *et al.*, 2000; Seo *et al.*, 1997).

IBV is highly variable and evolves rapidly by mutation and recombination (Cavanagh *et al.*, 1992; Kottier *et al.*, 1995; Lee & Jackwood, 2000), leading to the continuous emergence of new genetic and antigenic variants worldwide (genotypes and serotypes) (Gough *et al.*, 1992; Liu & Kong, 2004). However, few variants are able to persist for extended time periods and spread in new territories to become of evolutionary and economic importance. The monitoring of IBV populations from different geographical locations is important in order to map IBV genetic diversity and identify the origin and spreading of relevant genotypes.

Analyses of the S and N genes have been widely employed to identify IBV genotypes and explore phylogenetic and epidemiological evolution of IBV strains (Adzhar *et al.*, 1997; Kant *et al.*, 1992; Liu *et al.*, 2006). Most studies use for genotyping the coding region of the S1 subunit as the main inducer of protective immunity (de Wit *et al.*, 2011). Genetic analyses are also useful for the selection of the most appropriate vaccination programmes using attenuated and inactivated IBV strains (Farsang *et al.*, 2002), a particularly important issue because IBV serotypes have a low degree of cross-protection (Cavanagh, 2003). In several parts of the world, including most South American countries, a single strain type (Massachusetts) is included in the officially authorized vaccines, but additional strain types (e.g. Connecticut, Arkansas, D207, D3896, 4/91) are permitted elsewhere.

The origin, emergence and expansion of IBV genotypes within and across continents have not been previously analysed despite the virus’s relevance for the poultry industry and the evolutionary importance of coronavirus. Most studies deal with genetic variation in particular geographical regions or countries, without performing Phylodynamic analysis to explore the temporal behaviour of IBV (Han *et al.*, 2011; Liu *et al.*, 2006). IBV dynamics in South America is particularly poorly understood. The first IBV isolates (Massachusetts-type) were reported in Brazil in the 1950s (Hipólito, 1957) and in Chile in the 1970s (Hidalgo *et al.*, 1976). Other IBV genotypes have been identified in Brazil, Argentina and Colombia through sequence analysis of partial-length S1 coding region of different size and position (Alvarado *et al.*, 2005; Chacon *et al.*, 2011; Felippe *et al.*, 2010; Rimondi *et al.*, 2009; Villarreal *et al.*, 2007). However, the full-length S1 coding region and N gene in South American strains have not yet been analysed, and there are no comparative studies of the strains that are circulating among the countries. To our knowledge, the present study provides the first integrative analysis that focuses on South American IBV strains, their phylogenetic relationships with genotypes circulating worldwide and their emergence and expansion in the continent.

A total of 24 samples were collected from different outbreaks in commercial broilers with respiratory signs, during 2009–2012 in Uruguay and Argentina (Table S1, available in the online Supplementary Material). IBV presence was confirmed by real-time reverse transcriptase PCR (RT-PCR) (Callison *et al.*, 2006). Tissues were processed as described by Rimondi *et al.* (2009). The complete S1 coding region and N gene were obtained by RT-PCR using new or previously described primers (Table S2) and standard conditions (Hernández *et al.*, 2006).

A full-length S1 dataset was built using the nucleotide sequence of the S1 subunit (position from ATG start codon to the cleavage recognition site) of global reference strains (*n* = 30), the Argentine and Uruguayan strains here obtained (*n* = 24), and Brazilian IBV sequences (*n* = 19) that were recently deposited in GenBank. Partial-length S1 datasets were built using different portions of the S1 coding region. A full-length N gene dataset was built using global reference strains (*n* = 24), and the Argentine and Uruguayan strains here obtained (*n* = 24).

Sequences were aligned using mafft (Katoh *et al.*, 2002), and the best-fit model of nucleotide substitution was selected under the Akaike information criterion and Bayesian information criterion as implemented in jModelTest (Posada, 2008). Maximum-likelihood trees were inferred using PhyML (Guindon & Gascuel, 2003). Phylogenetic trees were visualized and edited with TreeGraph2 (Stöver & Müller, 2010).

For Phylodynamic analysis, two genotype-specific datasets were built with partial-length S1 sequences (positions 1–528); partial sequences were used in order to include all South American strains available in GenBank. The Bayesian skyline plot model implemented in beast v.1.7.5 was used for estimating population parameters without a prior function for demographic dynamics (Drummond *et al.*, 2012). Four independent Markov chain Monte Carlo runs were performed using the HKY+I+G model with base frequencies estimated from the data. A chain length of 200 000 000 with a burn in consisting of 2 000 000 steps was enough to ensure convergence, as evidenced by effective sample size values higher than 200 for each sampled parameter. Plots were generated using in-house R scripts.

The full-length S1 coding region and N gene were amplified and sequenced in all samples. In the phylogenetic analysis based on the S1 coding region, the South American strains group in three well-supported clades that we here denoted South America I (SAI), Asia/South America II (A/SAII) and Massachusetts-like genotypes (Fig. 1).

**Fig. 1.**
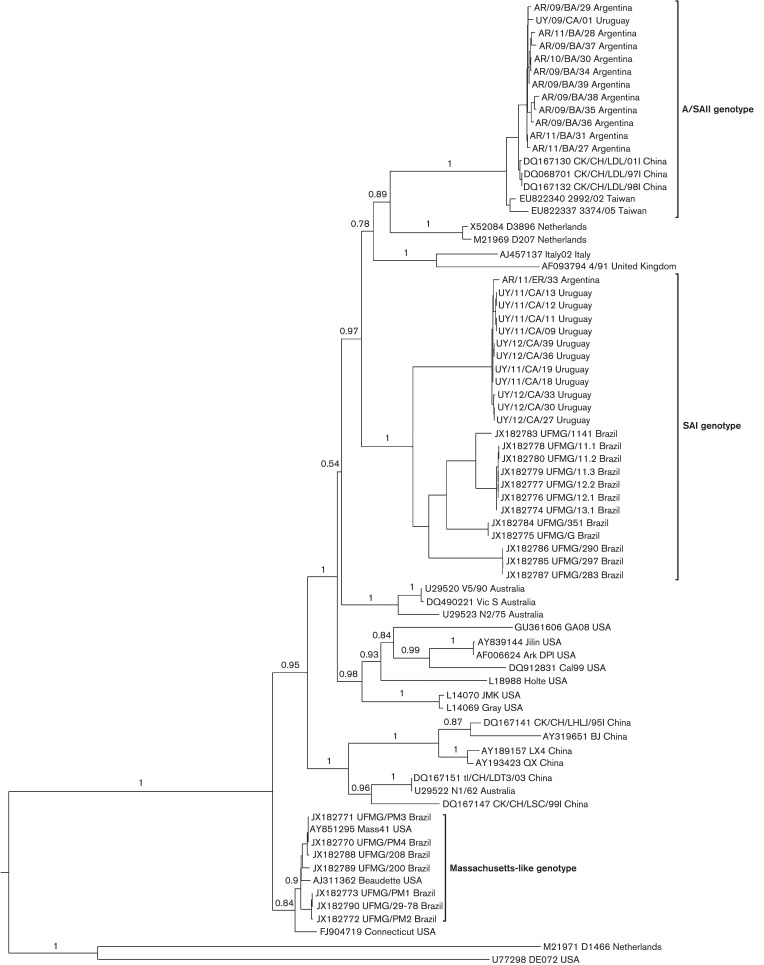
Phylogenetic tree inferred by using the maximum-likelihood method with GTR+I+G substitution model. Phylogenetic reconstruction was carried out using the full-length S1 coding region of South American IBV strains and reference strains. Mapping uncertainties for internal nodes are shown as approximate likelihood ratio test values.

The SAI genotype comprises 24 Argentine, Brazilian and Uruguayan strains. The nucleotide and amino acid identities within this genotype vary from 86.9 to 100 % and from 83.9 to 99.9 %, respectively. The time to the most recent common ancestor (tMRCA) of the SAI genotype was 48 years (Fig. 2). Accordingly, the SAI genotype is an exclusive South American lineage that emerged in approximately 1964 (Figs. 1 and 2). Strains of the SAI genotype would have been co-circulating with Massachusetts-like strains that have been reported in South America since the 1950s (Hidalgo *et al.*, 1976; Hipólito, 1957). The SAI genotype was extremely successful and spread in most South American territories (Argentina, Brazil and Uruguay).

**Fig. 2.**
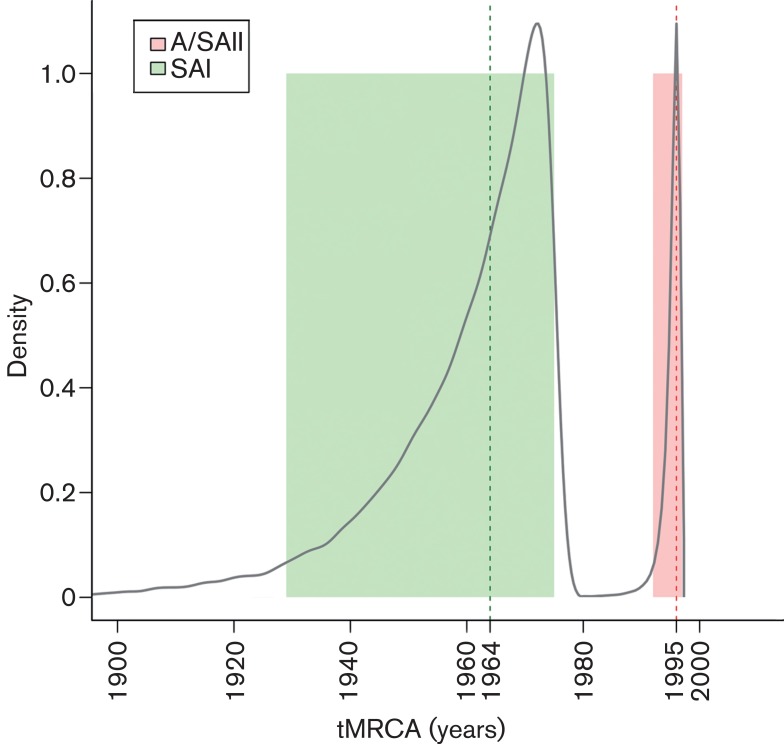
Posterior probability densities for the tMRCA inferences for SAI and A/SAII genotypes. The SAI genotype dataset (*n* = 81) comprised strains collected from 1975 to 2012, and the A/SAII genotype dataset (*n* = 25) comprised strains collected from 1996 to 2011. Vertical dashed lines represent the mean estimated time for each ancestor and coloured boxes reflect the 95 % high probability density values.

The A/SAII genotype comprises most Argentine, one Uruguayan, and Chinese and Taiwanese strains. The nucleotide and amino acid identities vary from 97.9 to 99.9 % and from 95.1 to 100 %, respectively. With respect to the SAI genotype, nucleotide identities vary from 79.3 to 83.3 % and amino acid identities vary from 71.6 to 78.1 %. The Asiatic variants of the A/SAII genotype belong to the recently described genogroup denoted the CK/CH/LDL/97I-type (Han *et al.*, 2011). Strains of this genogroup were first detected in China in 1996 (Yu *et al.*, 2001), and were systematically isolated in Asia during 1996–2002 associated with proventriculitis and nephritis (Huang *et al.*, 2004; Liu *et al.*, 2009a). According to the estimates of the tMRCA, the A/SAII genotype emerged in approximately 1995 (Fig. 2). This date is very close to the first detection of this genotype in Asia but long before the first record in South America (2006). This fact and the basal location of Asiatic strains in the phylogenetic tree (Fig. 1) support that the A/SAII originated in Asia and was later introduced into South America. Although there is evidence of the spread of IBV strains among close geographical regions (Ignjatovic *et al.*, 2006; Ignjatović & Sapats, 2000; Meulemans *et al.*, 2001), the long-distance intercontinental dispersion is unusual. One possible way could be through the commercial trade between countries (Cobb, 2011; Hosseini *et al.*, 2010). However, there is no official record of 1-day-old chicks or other poultry product trade from Asia to South America. Alternatively, the introduction of the Asian strain could have taken an indirect route, as supported by the recent detection of this genotype in Italy (Toffan *et al.*, 2013). The role of birds in dissemination of the new IBV variants still remains unclear. Even though wild species can be IBV carriers, there is no evidence that IBV strains infected migratory birds and were transmitted over long distances as occurs with influenza virus (Brown *et al.*, 2006).

The Massachusetts-like genotype includes seven Brazilian field strains and Massachusetts-type vaccine strains, and has nucleotide and amino acid identity values varying from 97.5 to 99.9 %, and from 95.8 to 99.8 %, respectively. The sequence similarity is less than 79 % with respect to the SAI and A/SAII genotypes.

The phylogenetic analysis based on partial-length S1 sequences shows that the SAI genotype was not detected outside Argentina, Brazil and Uruguay, and that the strains of the A/SAII genotype seem to be circulating in Chile and Colombia but not in Brazil. Vaccine-type strains (Massachusetts, Connecticut and Arkansas) were detected in Brazil, Argentina and Colombia. Some field strains could not be assigned to the SAI and A/SAII genotypes or to any vaccine-type and might represent novel South American genotypes (Table S3). As previous studies in South America did not analyse full-length S1 sequences, the comparison between strains and the detection of new genotypes is severely hindered. Analyses of different parts of S1 may also result in distinct levels of homology, leading to misinterpretation of the relationship between virus strains (de Wit *et al.*, 2011), and could avoid the identification of recombinant S1 sequences (Dolz *et al.*, 2008; Jia *et al.*, 1995; Wang *et al.*, 1993). As we obtained the complete S1 coding region, it was possible to make comparisons with partial sequences from other countries to describe a more comprehensive scenario of the South American IBV variability, highlighting the benefits of characterizing complete sequences rather than focusing on smaller coding regions.

The presence of relevant amino acid differences in S1 sequences between the SAI and A/SAII genotypes also supports their different ancestral origins. The A/SAII genotype has a characteristic deletion of three amino acid residues (T/NGP), with respect to the SAI genotype, within the second hypervariable region of the S protein. Insertions and deletions in S1, particularly in the hypervariable regions, are described frequently and have an important role in the generation of IBV genetic diversity (Abro *et al.*, 2012; Liu *et al.*, 2009a). The SAI genotype has the most common cleavage recognition site sequence (Arg-Ser/Leu-Arg-Arg). Both Asiatic and South American A/SAII strains share a distinctive and exclusive cleavage recognition site (Arg-Thr-Gly-Arg) with yet-unknown functional implications (Jackwood *et al.*, 2001).

In the phylogenetic analysis based on the full-length N gene, the SAI and South American A/SAII strains form a well-supported monophyletic group (Fig. 3). The nucleotide and amino acid identity vary values from 95.9 to 100 % and from 95.3 to 100 %, respectively. The South American and the Asiatic strains of the A/SAII genotype do not cluster together, revealing a phylogenetic incongruence. This differential clustering suggests that, after the Asian strain introduction, the A/SAII genotype underwent a recombination event that transferred the N gene of the SAI genotype to the Asiatic strains. Recombination events are frequently described in IBV field strains (Dolz *et al.*, 2008; Lee & Jackwood, 2000) as a consequence of the large genome size, a replication machinery that dissociates and reassociates from the template RNA, and the availability of full-length and subgenomic-length strands for template switching (Fu & Baric, 1992).

**Fig. 3.**
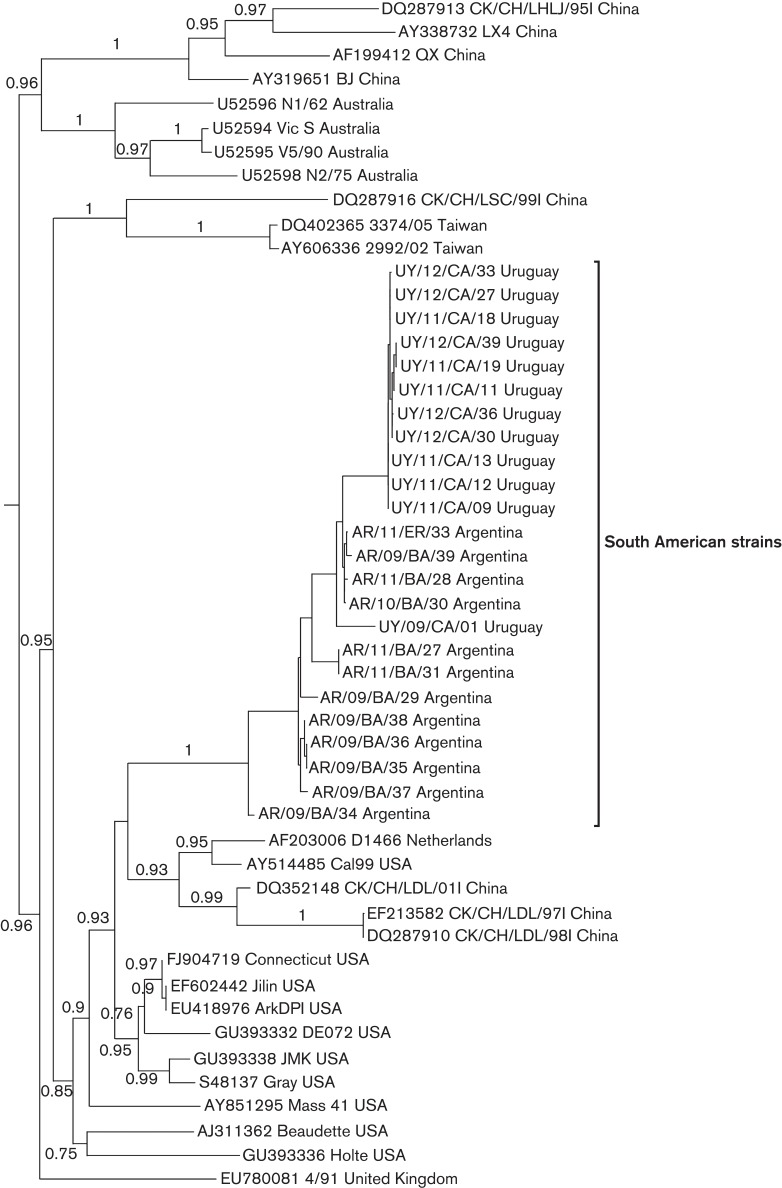
Phylogenetic tree inferred by using the maximum-likelihood method with GTR+I+G substitution model. Phylogenetic reconstruction was carried out using the full-length N gene sequence of South American IBV strains and reference strains. Mapping uncertainties for internal nodes are shown as approximate likelihood ratio test values.

The SAI and A/SAII genotype strains clearly differ from the Massachusetts strains that are included in the vaccine formulations of most South American countries (Figs. 1 and 3). The Massachusetts-like genotype clusters separately in the phylogenetic tree and shows low nucleotide and amino acid similarity with the SAI and A/SAII genotypes in both the S1 and N sequences, suggesting a limited level of cross-protection.

Conventional serotype testing and *in vivo* protection studies have been performed between strains belonging to the Massachusetts and South American genotypes. Brazilian strains, here assigned to the SAI genotype, and Asiatic strains of the A/SAII genotype were not completely neutralized by antisera specific to a Massachusetts strain (Chacon *et al.*, 2011; Liu, *et al.*, 2009b; Yu *et al.*, 2001). However, the differences in the N gene of Asiatic and South American strains of A/SAII genotype (Fig. 3) open the possibility of a dissimilar antigenic behaviour.

The inadequate immune response provided by Massachusetts strain vaccination may have resulted in the high substitution rates here observed, 5.34×10^−3^ and 1.81×10^−3^ nucleotides per site per year for the SAI and A/SAII genotypes, respectively. Intermediate or low levels of immunity result in the highest rate of emergence of viral variants because solid immunity severely limits virus replication and, thus, the generation of genetic variants (Toro *et al.*, 2012). In the absence of a specific vaccine, the substitution rate of the 793/B-type IBV was reported as 3×10^−3^ nucleotides per site per year (Cavanagh *et al.*, 1998). On the other hand, evaluation of Massachusetts and Connecticut strains collected over 41 and 25 years, indicated that substitution rates ranged from 10^−4^ to 10^−6^ nucleotides per site per year, where attenuated live vaccines of these genotypes were routinely used (McKinley *et al.*, 2011).

Together, our results reveal that the dynamic in South America is unusual as it involves two main genotypes with different evolutionary histories that have persisted in the continent for several years and acquired a notorious wide geographical distribution. The ability of the SAI and A/SAII genotypes to evade the immune response of Massachusetts-type vaccines may explain their successful spreading in all South American countries.

## Supplementary Data

26300 Supplementary Data
