# Supplementary material for: Phylodynamic analysis of avian infectious bronchitis virus in South America
Source: J Gen Virol. 2015 Jun;96(Pt 6):1340–6. doi: 10.1099/vir.0.000077 (PMC7081071; doi:10.1099/vir.0.000077)
Supplement: Supplementary file 1 — Supplementary Data [file jgv-96-1340-s001.pdf]

Supplementary material

Article title: Phylodynamics analysis of avian infectious bronchitis virus in South America

Author names: Ana Marandino, Ariel Pereda, Gonzalo Tomás, Martín Hernández, Gregorio Iraola, María Isabel Craig, Diego Hernández, Alejandro Banda, Pedro Villegas, Yanina Panzera, Ruben Pérez

Affiliation of the corresponding autor: Sección Genética Evolutiva, Instituto de Biología, Facultad de Ciencias, Universidad de la República, Iguá 4225, 11400 Montevideo, Uruguay.

E-mail of the corresponding author: rperez@fcien.edu.uy

Table S1. Argentine and Uruguayan strains employed in evolutionary studies, according to the year of isolation, accession number and genotype. (SAI) South America I. (A/SAII) Asia/South America II (A/SAII).

**Isolate designation**	**Year of isolation**	**Accession number (S1)**	**Accession number (N)**	**Genotype**
UY/09/CA/01*	2009	KM658245	KM658209	A/SAII
AR/09/BA/29	2009	KM658246	KM658221	A/SAII
AR/09/BA/34	2009	KM658247	KM658222	A/SAII
AR/09/BA/35	2009	KM658248	KM658223	A/SAII
AR/09/BA/36	2009	KM658249	KM658224	A/SAII
AR/09/BA/37	2009	KM658250	KM658225	A/SAII
AR/09/BA/38	2009	KM658251	KM658226	A/SAII
AR/09/BA/39	2009	KM658252	KM658227	A/SAII
AR/10/BA/30	2010	KM658253	KM658228	A/SAII
UY/11/CA/09	2011	KM658233	KM658210	SAI
UY/11/CA/11	2011	KM658234	KM658211	SAI
UY/11/CA/12*	2011	KM658235	KM658212	SAI
UY/11/CA/13	2011	KM658236	KM658213	SAI
UY/11/CA/18	2011	KM658237	KM658214	SAI
UY/11/CA/19	2011	KM658238	KM658215	SAI
AR/11/BA/27	2011	KM658254	KM658230	A/SAII
AR/11/BA/28	2011	KM658255	KM658231	A/SAII
AR/11/BA/31	2011	KM658256	KM658229	A/SAII
AR/11/ER/33*	2011	KM658244	KM658232	SAI
UY/12/CA/27	2012	KM658239	KM658216	SAI
UY/12/CA/30	2012	KM658240	KM658217	SAI
UY/12/CA/33	2012	KM658241	KM658218	SAI
UY/12/CA/36	2012	KM658242	KM658219	SAI
UY/12/CA/39	2012	KM658243	KM658220	SAI

*These samples were inoculated in specific pathogen-free chicken embryos

Table S2. Primes used in this study.

Primer	Sequence 5´→ 3´	Polarity	Position*	Amplicon size
IBV-S1	ACTGAACAAAAGACAGACTT	+	20313-20332	783
IBV-S2	CCATCTGAAAAATTRCCAGT	-	21076-21095	
IBV-S5	TTTGTYAATGGTACTGYACA	+	21001-21020	1027
IBV-S4	CATAACTAACATAAGGGCAA	-	22008-22027	
IBV-N2**	GTCTTGTCCCGCGTGTA	+	25846-25862	437
IBV-N1**	ACCCTTACCAGCAACCC	-	26267-26283	
IBV-N3	GATGGTATAGTGTGGGTTGCTG	+	26254-26275	971
IBV-N4	TTCCCTGGCGATAGACATGTA	-	27205-27224	

* Oligonucleotide position according to IBV strain M41 sequence (AY851295)

** Zwaagstra et al. (1992)

Table S3. Putative South American genotypes inferred with maximum-likelihood trees based on different portions of the S1 coding region (positions: 1-528, 229-528, 1-310, 134-722 and 740-1089) of all South American strains available in GenBank, including Brazilian (n=91), Argentine (n=19), Colombian (n=17) and Chilean (n=9) strains, and the obtained in the present study. South American genotype or cluster name assigned in original publication are shown in parentheses.

**S1 region analyzed***	**Collection year**	**Country**	**Putative South American genotypes**	**Reference**
1-528	1988-2000	Brazil	SAI<br>Massachusetts<br>Connecticut<br>Arkansas	Unpublished data (direct submission)
1-571	2002-2006	Brazil	SAI (Brazilian strains group)	Villarreal et al., 2007
229-556	2003-2009	Brazil	SAI (Cluster D207)<br>Massachusetts<br>Connecticut	Felippe et al., 2010
1-536	2003-2009	Brazil	SAI (Genotype BR I)	Chacón et al., 2011
134-722	2001-2008	Argentina	SAI (Cluster C)<br>A/SAII (Cluster A)<br>(Cluster B)<br>Massachusetts<br>Connecticut	Rimondi et al., 2009
1 – 310	2003	Colombia	A/SAII (Genotype C)<br>(Genotype A)<br>(Genotype B)<br>(Genotype D)<br>Massachusetts<br>Connecticut	Alvarado et al., 2005
740-1089	2008-2009	Chile	A/SAII	Unpublished data (direct submission)

Supplementary references

Alvarado, A. I. R., Villegas, P., Mossos, N., & Jackwood, M. W. (2005). Molecular Characterization of Avian Infectious Bronchitis Virus Strains Isolated in Colombia During 2003. *Avian Diseases* ***49*****(4),** 494–499.

Chacon, J. L., Rodrigues, J. N., Assayag Junior, M. S., Peloso, C., Pedroso, A. C., & Ferreira, A. J. P. (2011). Epidemiological survey and molecular characterization of avian infectious bronchitis virus in Brazil between 2003 and 2009. *Avian Pathology* ***40*****(2),** 153–162.

Felippe, A. P. A. N., Silva, L. H. A., Santos, M. M. A. B., Spilki, F. R., & Arns, C. W. (2010). Genetic Diversity of Avian Infectious Bronchitis Virus Isolated from Domestic Chicken Flocks and Coronaviruses from Feral Pigeons in Brazil Between 2003 and 2009. *Avian Disease* ***54*****(4),** 1191–1196.

Rimondi, A., Craig, M. I., Vagnozzi, A., König, G., Delamer, M., & Pereda, A. (2009). Molecular characterization of avian infectious bronchitis virus strains from outbreaks in Argentina (2001-2008). *Avian Pathology* ***38*****(2),** 149–153.

Villarreal, A. L. Y. B., Brandão, P. E., Chacón, J. L., Saidenberg, A. B. S., Assayag, M. S., Ferreira, A. J. P., Jones, A. R. C. (2007). Molecular Characterization of Infectious Bronchitis Virus Strains Isolated from the Enteric Contents of Brazilian Laying Hens and Broilers. *Avian Diseases* ***51*****(4),** 974–978.

Zwaagstra, K., van Der Zeijst, B., Austers, J. (1992). Rapid detection and identification of avian infection bronchitis virus. *Journal of Clinical Microbiology* ***30(1),*** 79–84.
